# Mechanism based therapies enable personalised treatment of hypertrophic cardiomyopathy

**DOI:** 10.1038/s41598-022-26889-2

**Published:** 2022-12-28

**Authors:** Francesca Margara, Yiangos Psaras, Zhinuo Jenny Wang, Manuel Schmid, Ruben Doste, Amanda C. Garfinkel, Giuliana G. Repetti, Jonathan G. Seidman, Christine E. Seidman, Blanca Rodriguez, Christopher N. Toepfer, Alfonso Bueno-Orovio

**Affiliations:** 1 Department of Computer Science, University of Oxford, Oxford, UK; 2 Radcliffe Department of Medicine, Division of Cardiovascular Medicine, University of Oxford, Oxford, UK; 3 Department of Genetics, Harvard Medical School, Boston, MA USA; 4 Cardiovascular Division, Brigham and Women’s Hospital, Boston, MA USA; 5 Howard Hughes Medical Institute, Chevy Chase, MD USA; 6 Wellcome Centre for Human Genetics, University of Oxford, Oxford, UK

**Keywords:** Cardiomyopathies, Virtual screening, Computer modelling, Induced pluripotent stem cells

## Abstract

Cardiomyopathies have unresolved genotype–phenotype relationships and lack disease-specific treatments. Here we provide a framework to identify genotype-specific pathomechanisms and therapeutic targets to accelerate the development of precision medicine. We use human cardiac electromechanical in-silico modelling and simulation which we validate with experimental hiPSC-CM data and modelling in combination with clinical biomarkers. We select hypertrophic cardiomyopathy as a challenge for this approach and study genetic variations that mutate proteins of the thick (*MYH7*^R403Q/+^) and thin filaments (*TNNT2*^R92Q/+^, *TNNI3*^R21C/+^) of the cardiac sarcomere. Using in-silico techniques we show that the destabilisation of myosin super relaxation observed in hiPSC-CMs drives disease in virtual cells and ventricles carrying the MYH7^R403Q/+^ variant, and that secondary effects on thin filament activation are necessary to precipitate slowed relaxation of the cell and diastolic insufficiency in the chamber. In-silico modelling shows that Mavacamten corrects the MYH7^R403Q/+^ phenotype in agreement with hiPSC-CM experiments. Our in-silico model predicts that the thin filament variants TNNT2^R92Q/+^ and TNNI3^R21C/+^ display altered calcium regulation as central pathomechanism, for which Mavacamten provides incomplete salvage, which we have corroborated in TNNT2^R92Q/+^ and TNNI3^R21C/+^ hiPSC-CMs. We define the ideal characteristics of a novel thin filament-targeting compound and show its efficacy in-silico. We demonstrate that hybrid human-based hiPSC-CM and in-silico studies accelerate pathomechanism discovery and classification testing, improving clinical interpretation of genetic variants, and directing rational therapeutic targeting and design.

## Introduction

Genetic diseases lack targeted and disease-specific treatment options that therapeutically address causal disease mechanisms^1^. This is complicated by unresolved disease pathophysiology and population heterogeneity. Human-based modelling and simulation studies address these challenges by uncovering mechanisms bridging from mutation to clinical biomarkers, in combination with experimental and clinical data^2^. Thus, they can unravel insights into key features of disease pathomechanisms, improving therapeutic development and supporting precision medicine in the clinic.

Among genetic diseases, hypertrophic cardiomyopathy (HCM) is a common condition. Affecting approximately 1 in 500 people, HCM can cause arrhythmias, heart failure, and sudden cardiac death in affected individuals^3^. HCM is caused predominantly by mutations in sarcomere genes^4^, most abundantly in *MYH7*, *MYBP3*, *TNNT2,* and *TNNI3*. Within each of these genes there are many possible causative variants of HCM, with diverse pathomechanisms. Establishing genotype–phenotype relationships and pathomechanisms in HCM is the main bottleneck to clinical translation and precision medicine. This is even with the implementation of novel technologies such as CRISPR/Cas-9, human induced pluripotent stem cell-derived cardiomyocytes (hiPSC-CMs), and cutting-edge protein expression systems, which have allowed more rapid phenotyping.

Most HCM variants fall within genes that encode proteins of the cardiac sarcomere, which can be robustly modelled in-silico^5^. Human in-silico models enable simulation of force generation by calcium regulation of the troponin complex and the cyclical interactions between myosin and actin^6^. This is important as altered force at the myofilament can drive pathogenesis of many forms of cardiomyopathy, and novel therapeutics aim to modulate cardiac contractility for treating these therapeutically orphan heart diseases^7^. Human-based in-silico frameworks twinned with hiPSC-CM data can be leveraged to simulate single cell function and scale to entire chambers of the heart^8^ to accelerate phenotyping and drug discovery.

Previous computational studies of HCM have investigated disease pathomechanisms from the molecular dynamics to the cellular and organ levels^9^. These studies have unravelled important insights linking mutations in sarcomere genes to changes in contractility and arrhythmogenesis^10–13^, providing predictions of key mechanisms that underlie arrhythmia in HCM whilst explaining the efficacy of pharmacological targets^14^. Whole organ studies enabled the connection between heterogeneous cardiac substrates, the electrocardiogram, and arrhythmic risk^15–17^. Building on this, we present human modelling and simulation that extends this prior work by bridging disease-causing mutations to clinical disease. Our study outlines a hybrid biological and in-silico framework to accelerate hypothesis generation and testing in variant classification, pathomechanism discovery, and therapeutic targeting. We integrate molecular changes, with cellular level phenotypes, which are scaled to the whole organ. We do this to establish and test genotype–phenotype relationships to identify therapeutic targets and accelerate precision medicine.

**Figure 1:**
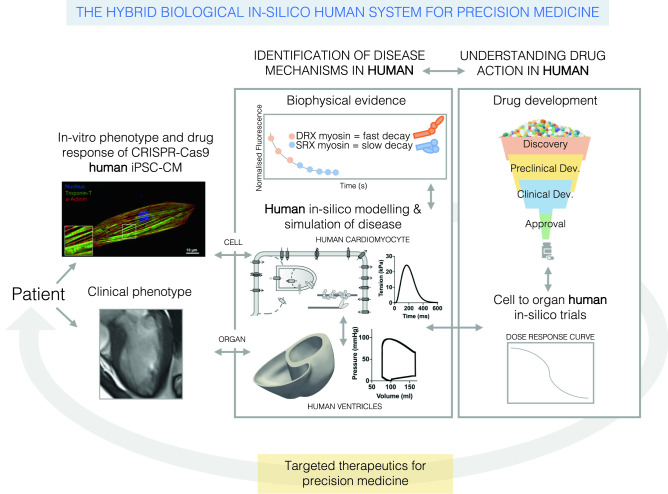
The hybrid biological and in-silico human system defined in this work to synergistically accelerate hypothesis generation and testing in variant classification, pathomechanism discovery, and therapeutic targeting. From patient genotype, hiPSC-CMs can be generated via CRISPR-Cas9 to express patient’s genetic variant. Cellular phenotypes and responses to pharmaceutical interventions can be studied in-vitro. Based on this and available biophysical evidence on mutational protein function, hypotheses on variant-specific disease mechanisms can be formulated. These are tested in in-silico human models of cardiac electromechanical function, from cell to organ. In-silico human disease models, informed by clinical data, enable simulation of the multiscale features of disease phenotypes. In-silico drug trials can be conducted to test the safety and efficacy of pharmacological therapies on specific disease phenotypes and pathomechanisms. Results from in-silico trials can be integrated into the drug development process. In-silico trials can also identify new therapeutic targets and provide evidence to inform the best targeted therapeutic approach for the individual patient.

We therefore use a human in-silico modelling and simulation framework integrated with CRISPR/Cas-9 and hiPSC-CM modelling of disease, biophysical and clinical data, to accelerate biological and clinical investigation (Fig. 1). Our in-silico models have previously been extensively calibrated and validated to human data^18–21^. CRISPR/Cas-9-edited hiPSC-CM models of thick filament HCM have previously shown consistency with in-vivo models of HCM, human tissue samples, and clinical HCM cohort data^22,23^. Our hybrid human-based framework defines novel disease pathomechanisms across diverse HCM-causative genetic variants. We use this information to predict and explain divergent efficacy of Mavacamten^24,25^ (now commercially marketed as Camzyos), a first-in-class HCM-targeted small molecule, across HCM genotypes. We provide digital evidence of mechanistic pharmacological targets that can be used to tailor genotype-specific phenotype correction, as validated with hiPSC-CMs. We propose this hybrid human-based framework can be extended widely within cardiovascular medicine to accelerate precision medicine.

## Results

### Simulating human HCM defines key mechanistic differences between *MYH7*, *TNNT2*, and *TNNI3* variants

Similar contractile profiles are observed in hiPSC-CMs expressing different HCM genetic variants across the *MYH7* ($\upbeta$-myosin heavy chain), *TNNT2* (troponin T), and *TNNI3* (troponin I) genes. A phenotype of hypercontractility and slowed relaxation is reported for the *MYH7*^R403Q/+^, *TNNT2*^R92Q/+^, and *TNNI3*^R21C/+^ variants^22,26–28^, which occurs alongside prolonged calcium decay times, and a faster time to transient peak observed in *TNNI3*^R21C/+^^26–28^. hiPSC-CM contractile profiles are consistent with those obtained in mouse models of disease^22,23^.

We conducted human simulation studies to determine the mechanistic pathways that explain these similar cellular phenotypes, considering their diverse clinical manifestations. We generated populations of in-silico virtual cardiomyocytes using our extended cellular electromechanical model of human cardiomyocyte electromechanical function^20^ (Supplementary Figs. 1 and 2). This model was previously constructed, calibrated, and extensively validated with human cellular data^18–20^. Within this model we integrated variant-specific biophysical evidence of pathogenesis. We based our hypotheses on both well-established and novel published reports on mechanisms of pathogenesis that considered a variety of experimental systems including human cardiac ventricular tissues from patients with HCM^22,29–32^. We compared our simulation results with experimental findings from hiPSC-CMs to extend the utility of these findings which are from an immature system to an adult in-silico model and see if the result holds true across systems. It is through the iterative exchange of experimental and model predictions that we can provide new insights on HCM.

#### MYH7^R403Q/+^ decreases myosin SRX increasing thin filament activation causing cellular HCM

**Figure 2:**
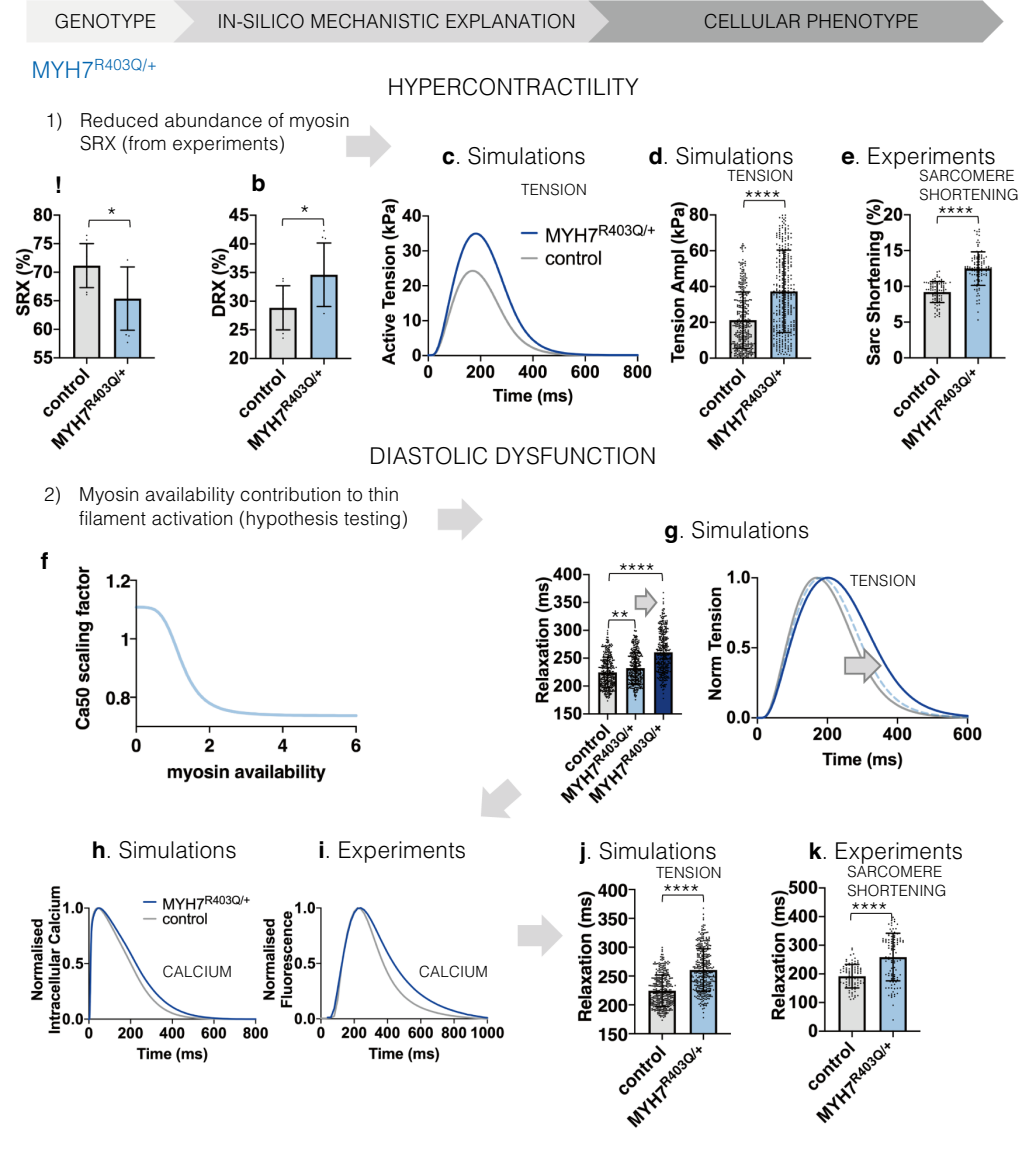
Mechanistic explanation of the MYH7^R403Q/+^ cellular phenotype of hypercontractility and diastolic dysfunction through human-based modelling and simulation informed by biophysical evidence. (**a**,**b**) Proportion of myosin heads in SRX and DRX conformations in WT and MYH7^R403Q/+^ hiPSC-CMs. Data are presented as mean and SD, significance was tested by two-tailed unpaired t test (N = 9 in control and N = 10 in MYH7^R403Q/+^). (**c**) Comparison of simulated active tension waveforms in control and under MYH7^R403Q/+^. (**d**,**e**) Simulated MYH7^R403Q/+^ cardiomyocytes with larger myosin availability develop higher tension amplitude compared to control (**d**) in line with experimental hiPSC-CM data (**e**) which present increased sarcomere shortening. Data are presented as mean and SD, significance was tested by two-tailed unpaired t test for experimental data (N = 92 in control and N = 109 in MYH7^R403Q/+^) and Mann Whitney test for simulation data (N = 348 in control and MYH7^R403Q/+^). (**f**) Hypothesis tested in simulations to explain the pathway behind impaired relaxation in MYH7^R403Q/+^, i.e. the myosin-based contribution to thin filament activation. The scaling factor of Ca50 is reported. Ca50 represents the calcium concentration at half maximal thin filament activation and has $\upmu$M unit. Therefore, a larger Ca50 value means lower calcium sensitivity and vice versa. (**g**) Effect of myosin contribution to thin filament activation on simulated active tension relaxation: relaxation times are prolonged (dark blue) compared to the absence of feedback (light blue). Data are presented as mean and SD, significance was tested by Kruskal–Wallis with post hoc Dunn correction (N = 348 in control and MYH7^R403Q/+^). (**h**,**i**) Simulation results (**h**) that consider the myosin contribution to thin filament activation replicate the prolongation of the calcium transient decay observed experimentally (**i**).Experimental data presented as average trace (average over N = 113 for WT and N = 128 for MYH7^R403Q/+^) and simulation data presented as trace of the baseline model. (**j**,**k**) The combination of larger myosin availability and the myosin-based contribution to thin filament activation leads to a prolongation in the relaxation of simulated active tension (**j**) similar to experimental data (**k**). Data are presented as mean and SD, significance was tested by two-tailed unpaired t test for experimental data (N = 92 in control and N = 109 in MYH7^R403Q/+^) and Mann Whitney test for simulation data (N = 348 in control and MYH7^R403Q/+^).

We generated in-silico models of *MYH7*^R403Q/+^ cardiomyocytes with experimentally-informed^22^ reduced myosin super relaxation (SRX) (Fig. 2a,b). The myosin SRX deficit results in higher tension amplitude than control cells in-silico (Fig. 2c,d), corroborated by increased sarcomere shortening in *MYH7*^R403Q/+^ hiPSC-CMs^22^ (Fig. 2e). Reduced myosin SRX increases the disordered relaxed (DRX) myosin conformation, which becomes available to form crossbridges and drives hypercontractility^22,23^.

Simulation results demonstrated a mechanistic link between myosin availability and hypercontractility in *MYH7*^R403Q/+^ (Fig. 2a–e), but not slowed cellular relaxation. We tested if increased myosin DRX availability could directly influence thin filament activation^33^ (Fig. 2f), and explain prolonged cellular relaxation. These simulations showed that myosin-based activation of the thin filament triggered slowed relaxation (Fig. 2g, darker colours). Thin filament activation by increased DRX myosin prolonged calcium transient (Fig. 2h,i) and active tension (Fig. 2j,k) decay in our in-silico models, as subsequently validated in *MYH7*^R403Q/+^ hiPSC-CMs (Fig. 2i,k).

#### TNNT2^R92Q/+^ drives HCM by altering tropomyosin positioning and increasing calcium sensitivity

**Figure 3:**
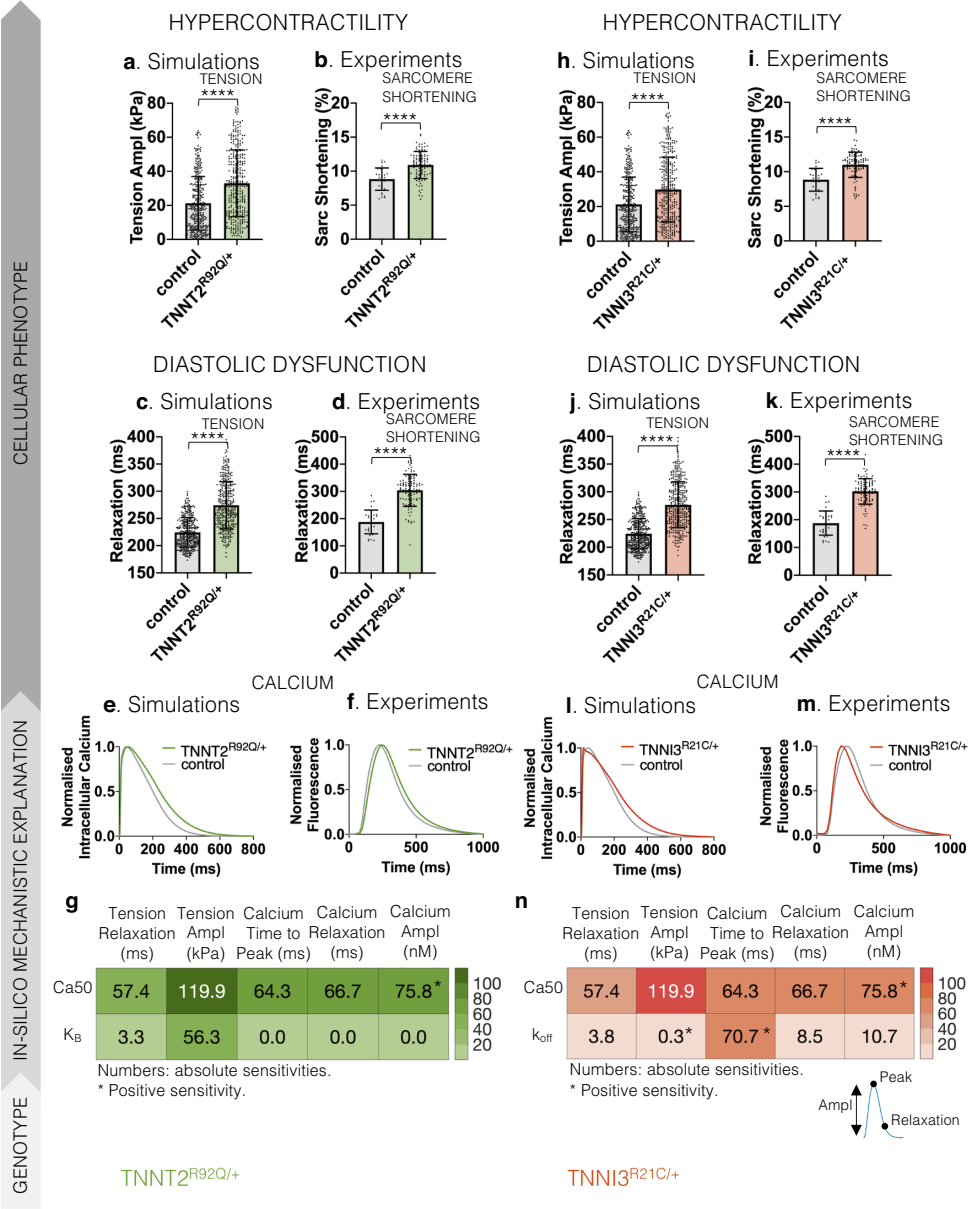
Mechanistic explanation of the TNNT2^R92Q/+^ and TNNI3^R21C/+^ cellular phenotypes of hypercontractility and diastolic dysfunction through human-based modelling and simulation informed by biophysical evidence. (**a**–**d**) Simulated TNNT2^R92Q/+^ cardiomyocytes, which have altered tropomyosin positioning (K_B_) and increased calcium sensitivity (Ca50), develop higher tension amplitude (Tension Ampl, **a**) with prolonged relaxation time (Relaxation, **c**) compared to control, in line with experimental hiPSC-CM data which present increased sarcomere shortening (**b**) and prolonged relaxation time (**d**). Data are presented as mean and SD, significance was tested with Mann Whitney test (N = 32 in control and N = 98 in TNNT2^R92Q/+^ for experimental data and N = 348 in control and TNNT2^R92Q/+^ for simulation data). (**e**,**f**) Simulation results (**e**) considering altered tropomyosin positioning and increased calcium sensitivity replicate the prolongation of the calcium transient decay observed experimentally in hiPSC-CMs (**f**). Experimental data presented as average trace (average over N = 113 for WT and N = 384 for TNNT2^R92Q/+^) and simulation data presented as trace of the baseline model. (**g**) Sensitivity analysis results. Absolute sensitivities of the active tension (Tension) and calcium transient (Calcium) biomarkers to changes in the model parameters that describe calcium sensitivity (Ca50) and tropomyosin positioning (K_B_) are reported. Biomarkers considered: Tension Relaxation denotes the time from tension peak to 90% decay, Tension Ampl denotes the difference between peak tension and baseline tension, Calcium Time to Peak denotes the time to reach peak calcium, Calcium Relaxation denotes the time from calcium peak to 90% decay, and Calcium Ampl denotes the difference between peak calcium and baseline calcium. (**h–k**) Simulated TNNI3^R21C/+^ cardiomyocytes, which have altered calcium sensitivity (Ca50) and dissociation rate from troponin (k_off_), develop higher tension amplitude (**h**) with prolonged relaxation time (**j**) compared to control, in line with experimental hiPSC-CM data which present increased sarcomere shortening (**i**) and prolonged relaxation time (**k**). Data are presented as mean and SD, significance was tested with Mann Whitney test (N = 32 in control and N = 89 in TNNI3^R21C/+^ for experimental data and N = 348 in control and TNNI3^R21C/+^ for simulation data). (**l**,**m**) Simulation results (**l**) considering altered calcium sensitivity and dissociation rate from troponin replicate the accelerated calcium rise and decelerated decay observed experimentally in hiPSC-CMs (**m**). Experimental data presented as average trace (average over N = 113 for WT and N = 60 for TNNI3^R21C/+^) and simulation data presented as trace of the baseline model. (**n**) Sensitivity analysis results. Absolute sensitivities of the active tension (Tension) and calcium transient (Calcium) biomarkers to changes in the model parameters that describe calcium sensitivity (Ca50) and calcium dissociation rate from troponin (k_off_) are reported.

Altered tropomyosin positioning^29^ and increased calcium sensitisation of the thin filaments^30,31^ are intrinsic biophysical defects in *TNNT2*^R92Q/+^. We wanted to establish if both mechanisms are necessary to observe the cellular phenotype. When both factors are considered in our in-silico *TNNT2*^R92Q/+^ cardiomyocytes, we observe an increased tension amplitude and prolonged tension decay, consistent with increased amplitude (Fig. 3a,b) and prolonged relaxation (Fig. 3c,d) of sarcomere shortening in *TNNT2*^R92Q/+^ hiPSC-CMs. Simulated *TNNT2*^R92Q/+^ cardiomyocytes also replicated the prolonged calcium transient decay observed in *TNNT2*^R92Q/+^ hiPSC-CMs (Fig. 3e,f).

We used sensitivity analysis to establish the individual contribution of the two biophysical mechanisms to the HCM phenotype. To describe tropomyosin positioning, we modified the model parameter for the reverse rate constant of the equilibrium constant (K_B_) between blocked and closed tropomyosin states^29^. To regulate myofilament calcium sensitivity, we varied the parameter Ca50 describing the calcium concentration at half maximal thin filament activation. We calculated the absolute sensitivity of active tension and the calcium transient to changes in K_B_ and Ca50 model parameters^34^ (Fig. 3g). Absolute sensitivities reflect changes in the phenotype (by means of the change in the biomarker considered) due to changes in the model parameter varied, where a higher value signifies a greater effect on the active tension or calcium transient for that specific model parameter. This allows the identification of the parameters that most affect or drive a specific phenotype. Ca50 had a larger impact on tension and calcium transient biomarkers compared to K_B_ (Fig. 3g). Both Ca50 and K_B_ contributed to changes in contractility (Fig. 3g, Tension Ampl). However, changes in tension and calcium relaxation were predominantly driven by changes in Ca50 (Fig. 3g, Tension Relaxation and Calcium Relaxation).

#### TNNI3^R21C/+^ drives HCM by altering calcium binding and dissociation from troponin

*TNNI3*^R21C/+^ hiPSC-CMs exhibit an accelerated calcium rise and slowed calcium decay, hypercontractility, and prolonged relaxation^26^. We investigated whether the cellular *TNNI3*^R21C/+^ phenotype can be explained by the altered binding of calcium to troponin observed in^32^ driven by altered interactions between the mutated troponin I and troponin C. We considered altered Ca50 at the thin filament, as well as slower calcium dissociation (k_off_) from troponin. These variables explained increased active tension amplitude and prolonged tension decay time in-silico, as respectively validated by increased amplitude (Fig. 3h,i) and prolonged relaxation (Fig. 3j,k) of sarcomere shortening in *TNNI3*^R21C/+^ hiPSC-CMs. Calcium transients of simulated *TNNI3*^R21C/+^ cardiomyocytes showed accelerated calcium rise and slowed decay, as observed in *TNNI3*^R21C/+^ hiPSC-CMs (Fig. 3l,m).

Sensitivity analysis explained the relative contributions of calcium sensitivity Ca50 and calcium dissociation k_off_ to the *TNNI3*^R21C/+^ phenotype. We computed the absolute sensitivities of the active tension and calcium transient biomarkers to changes in Ca50 and K_off_ model parameters^34^ (Fig. 3n) to evaluate which parameters were most important in determining the change in cellular phenotype. Ca50 has a larger impact on both active tension and calcium transient biomarkers than alterations of k_off_ (Fig. 3n). Alterations in k_off_ were key to replicating the *TNNI3*^R21C/+^ phenotype of accelerated calcium rise (Fig. 3n, Calcium Time to Peak).

### Human in-silico trials explain the cellular mechanisms of Mavacamten’s efficacy in thin and thick filament HCM

We used our in-silico findings to investigate pharmacological efficacy of targeted therapeutics in HCM. We performed in-silico trials to simulate the effect of Mavacamten on *MYH7*^R403Q/+^, *TNNT2*^R92Q/+^*,* and *TNNI3*^R21C/+^ variants. Mavacamten has been shown to improve cellular HCM phenotypes in *MYBPC3* and *MYH7* gene variants through stabilisation of myosin SRX^22,23^.

**Figure 4:**
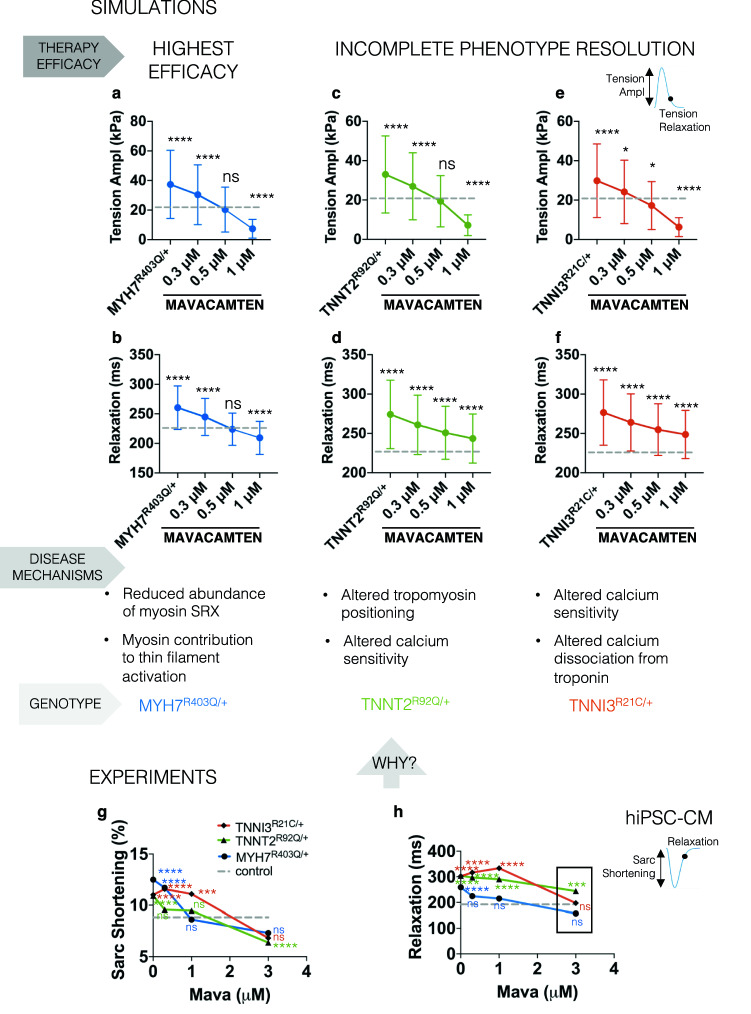
In-silico trials of Mavacamten on simulated MYH7^R403Q/+^, TNNT2^R92Q/+^, and TNNI3^R21C/+^ cardiomyocytes and comparison with experimental evidence. (**a**,**b**) Dose-dependent effect of Mavacamten on the active tension amplitude and relaxation time of simulated MYH7^R403Q/+^ cardiomyocytes, which are characterised by larger myosin availability due to a lower abundance of myosin SRX that contributes to thin filament activation. (**c**,**d**) Dose-dependent effect of Mavacamten on the active tension amplitude and relaxation time of simulated TNNT2^R92Q/+^ cardiomyocytes, which are characterised by altered tropomyosin positioning and calcium sensitivity. (**e**,**f**) Dose-dependent effect of Mavacamten on the active tension amplitude and relaxation time of simulated TNNI3^R21C/+^ cardiomyocytes, which are characterised by altered calcium sensitivity and dissociation rate from troponin. Simulation results recapitulated the experimentally observed responses to Mavacamten of hiPSC-CMs expressing the MYH7^R403Q/+^, TNNT2^R92Q/+^, and TNNI3^R21C/+^ variants (**g**,**h**) and provide an explanation based on mutation-specific disease mechanisms. All data presented in (**a**–**f**) is simulation data and is presented as mean and SD. Significance with respect to untreated control (dotted grey lines) was tested with Kruskal–Wallis with post hoc Dunn correction (N = 348 for each in-silico population). Data presented in (**g**,**h**) is experimental hiPSC-CM data and is presented as mean. Significance with respect to untreated control (dotted grey lines) was tested with Kruskal–Wallis with post hoc Dunn correction (N = 109 (mutant), N = 72 (mutant + 0.3 $\upmu$M Mava), N = 60 (mutant + 1 $\upmu$M Mava), and N = 60 (mutant + 3 $\upmu$M Mava) for MYH7^R403Q/+^; N = 98 (mutant), N = 106 (mutant + 0.3 $\upmu$M Mava), N = 98 (mutant + 1 $\upmu$M Mava), and N = 154 (mutant + 3 $\upmu$M Mava) for TNNT2^R92Q/+^; N = 89 (mutant), N = 128 (mutant + 0.3 $\upmu$M Mava), N = 52 (mutant + 1 $\upmu$M Mava), and N = 61 (mutant + 3 $\upmu$M Mava) for TNNI3^R21C/+^).

Mavacamten rescued both hypercontractility and impaired relaxation in *MYH7*^R403Q/+^ simulations. It dose-dependently reduced active tension amplitude of simulated *MYH7*^R403Q/+^ cardiomyocytes (Fig. 4a) by directly reducing myosin DRX, which deactivated knock-on thin filament activation in *MYH7*^R403Q/+^, showing correction of slowed relaxation (Fig. 4b). However, Mavacamten only partially corrected the contractile and prolonged relaxation phenotypes of *TNNT2*^R92Q/+^ (Fig. 4c,d) and *TNNI3*^R21C/+^ (Fig. 4e,f) cells. In our simulations Mavacamten can restore contractile function, irrespective of the mechanism that drives hypercontractility. However, correction of abnormal relaxation was only evident in models with overactive myosin, as found in *MYH7*^R403Q/+^. These in-silico predictions were borne out by our hiPSC-CM studies^22,26,27^ (Fig. 4g,h), where Mavacamten normalised cellular hypercontractility in all variants. However, it only corrected impaired relaxation at 3 µM in *TNNI3*^R21C/+^ and not at all for the *TNNT2*^R92Q/+^ variant, compared to 0.5 µM Mavacamten in *MYH7*^*403Q/*+^ hiPSC-CMs. Depression of contractility at 3 µM Mavacamten was marked in the *TNNI3*^R21C/+^ variant, suggesting this dosage may fall outside the therapeutic window. Discrepancies between quantitative model predictions (Fig. 4f) and experiments (Fig. 4h) can be explained considering (i) the differences between the systems (immature hiPSC-CMs versus adult in-silico cardiomyocytes), (ii) the difficulty in estimating the relationship between drug concentration and myosin heads in the dish, which then complicates the comparison between drug doses tested in-vitro and in-silico, and (iii) the effect of high concentrations of Mavacamten in suppressing the contractile transient which impairs the evaluation of the relaxation time (Supplementary Fig. 6). Nevertheless, the trend of responses to Mavacamten are similar for simulated cells and hiPSC-CMs providing confidence in model predictions.

### Human whole-ventricular electromechanical simulations of genotype-specific clinical phenotypes

**Figure 5:**
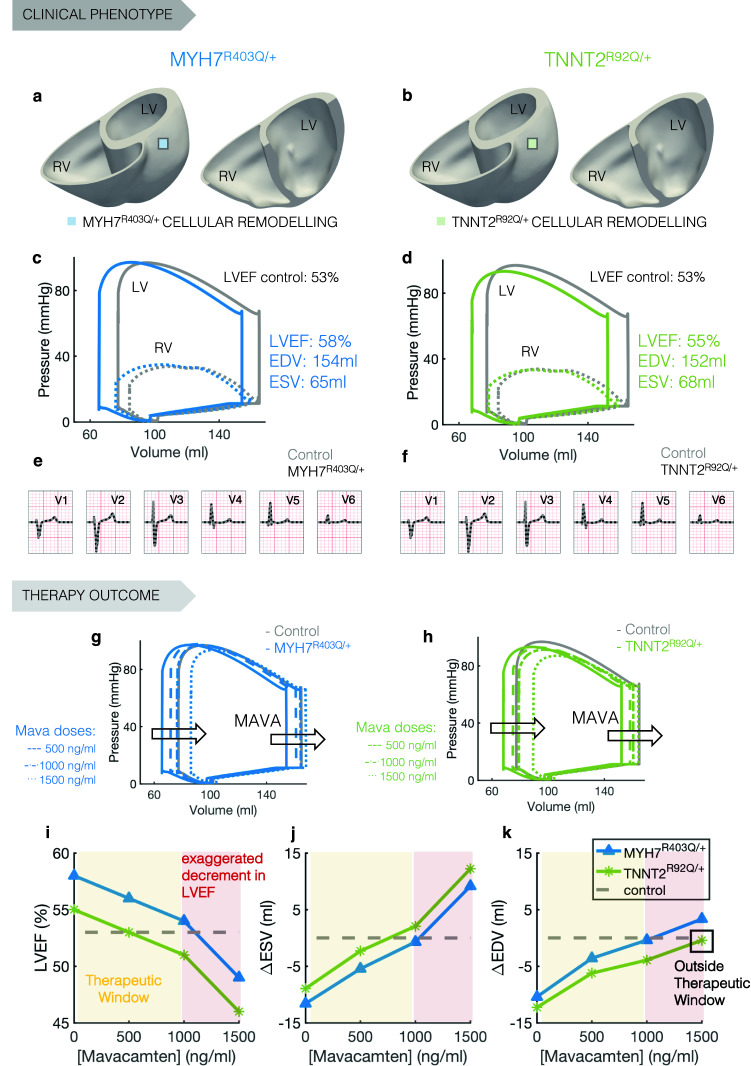
Human in-silico clinical trials of Mavacamten on genotype-positive phenotype-negative HCM virtual subjects. (**a**,**b**) Magnetic resonance derived anatomical model for the genotype-positive phenotype-negative HCM virtual subjects. Mutation-specific cellular remodelling is homogeneously distributed throughout the ventricles. (**c**,**d**) Pressure–volume loops of the left (solid lines) and right (dotted lines) ventricles of the virtual HCM subjects expressing the MYH7^R403Q/+^ (**c**, blue) and TNNT2^R92Q/+^ (**d**, green) genetic variants compared to control (grey). (**e**,**f**) Comparison of the 12 lead ECGs of the HCM subjects expressing the MYH7^R403Q/+^ (**e**) and TNNT2^R92Q/+^ (**f**) genetic variants compared to control (grey). (**g**,**h**) Dose-dependent effect of Mavacamten (500, 1000, and 1500 ng/ml) on the pressure–volume loop of the left ventricle of the HCM subjects expressing the MYH7^R403Q/+^ (**g**) TNNT2^R92Q/+^ (**h**) genetic variants. (**i**–**k**) Comparison of the dose-dependent effects of Mavacamten on the LVEF (**i**), end-systolic volume (**j**), and end-diastolic volume (**k**) of the HCM subjects expressing the MYH7^R403Q/+^ (blue) and TNNT2^R92Q/+^ (green) genetic variants. The yellow area represents the therapeutic window of the drug whereas the red area identifies drug concentrations that lead to a LVEF below 50%.

To bridge from bench to bedside, and test Mavacamten in a whole-organ in-silico clinical trial, we simulated the clinical phenotypes of two virtual genotype-positive phenotype-negative (i.e. before left ventricular (LV) hypertrophy) HCM subjects carrying the *MYH7*^R403Q/+^ and *TNNT2*^R92Q/+^ variants (Fig. 5a,b). We do this to separate and understand the disease pathomechanism in isolation from the downstream organ-level adaptations and disease progression pathways. Therefore, we used the same ventricular anatomy (chamber size and wall thickness) for the two virtual subjects and only varied cellular properties. We use *TNNT2*^R92Q/+^ virtual ventricles as an example of thin filament HCM where pathogenesis is predominantly driven by increased calcium sensitivity of myofilaments. As this is a common feature of *TNNT2* and *TNNI3* variants, as shown by our cellular studies, the results presented here for the *TNNT2* variant also holds for the *TNNI3* variant.

Human whole-ventricular electromechanical simulations showed that the *MYH7*^R403Q/+^ variant causes increased ventricular contractility, in good agreement with *MYH7*^R403Q/+^ cells (Fig. 5c). This is evident in the pressure–volume relation, driven by a smaller LV end-systolic volume in *MYH7*^R403Q/+^ of 65 ml versus 77 ml in healthy ventricles. LV end-diastolic volume was also reduced to 154 ml in *MYH7*^R403Q/+^ versus 165 ml in healthy ventricles. This signifies early-stage diastolic impairment. Overall, the LV ejection fraction (LVEF) increases in *MYH7*^R403Q/+^ compared to healthy control (58% vs 53%).

The *TNNT2*^R92Q/+^ variant virtual ventricles also displayed hypercontractility due to a smaller LV end-systolic volume (68 ml vs 77 ml of a healthy subject, Fig. 5d). LV end-diastolic volume was reduced to 152 ml in the *TNNT2*^R92Q/+^ versus 165 ml in healthy ventricles. The reduction in end-systolic volume was more pronounced in *MYH7*^R403Q/+^ than in *TNNT2*^R92Q/+^, while the reduction of end-diastolic volume was more pronounced in *TNNT2*^R92Q/+^ than in *MYH7*^R403Q/+^. Overall, *MYH7*^R403Q/+^ ventricles present a higher LVEF of 58% compared to the 55% of *TNNT2*^R92Q/+^ (Fig. 5c,d). Our model shows a stronger hypercontractility phenotype in *MYH7*^R403Q/+^, while *TNNT2*^R92Q/+^ ventricles exhibit a more severe diastolic dysfunction. In both cases, despite phenotypic ventricular changes in mechanical function typical of early genotype-positive phenotype-negative HCM^35^, simulated 12-lead ECGs did not show signs of abnormalities in the virtual patients (Fig. 5e,f) as occurs in the majority of preclinical subjects^36^. The absence of ECG changes induced by contractile dysfunction is an important insight for the understanding of the early changes of the disease.

### Mavacamten shows incomplete rescue of thin filament HCM in phenotype-negative virtual ventricles

Our cellular results demonstrate Mavacamten’s efficacy on variants that alter myosin SRX but suggest that variants not altering myosin availability (*TNNI3*, *TNNT2*) may receive a more modest cellular therapeutic benefit. We tested this hypothesis in a whole-organ in-silico clinical trial. We tested the effect of clinically-relevant plasma concentrations of Mavacamten at 500, 1000, and 1500 ng/ml on the electromechanical function of the *MYH7*^R403Q/+^ and *TNNT2*^R92Q/+^ virtual ventricles. We observed a dose-dependent correction of hemodynamics in *MYH7*^R403Q/+^ and *TNNT2*^R92Q/+^ carriers (Fig. 5g,h). This translated into a dose-dependent reduction of LVEF, which remained above the safety threshold (LVEF > 50%) up to 1000 ng/ml Mavacamten (Fig. 5i). Concentrations above 1000 ng/ml caused an exaggerated reduction in LVEF that may not be clinically desirable^37^. We observed a marked reduction of LVEF from 54 to 49% in *MYH7*^R403Q/+^ and from 51 to 46% in *TNNT2*^R92Q/+^ ventricles, in the 1000–1500 ng/ml Mavacamten range. Our results support the safety of Mavacamten when administered in the therapeutic range of 350–700 ng/ml in line with clinical findings^25^. Importantly, simulation results highlight differences in therapeutic efficacy. The abnormal end-systolic volume is dose-dependently corrected within the therapeutic window in both virtual variant carriers (Fig. 5j), but Mavacamten only restored the end-diastolic volume within the therapeutic window of *MYH7*^R403Q/+^ but not *TNNT2*^R92Q/+^ ventricles (Fig. 5k), in agreement with our cellular results. An appropriate dose of Mavacamten should be administered so that EDV is not increased beyond control values.

Altogether, our organ simulations underline the efficacy of Mavacamten for genetic variants that alter myosin conformations, such as *MYH7*^R403Q/+^, but further suggest an unmet need in correction of cell to organ abnormalities caused by thin filament HCM, which we investigate below.

### Human in-silico trials identify pathomechanism-targeted thin filament HCM therapeutics

Given the incomplete correction of HCM cellular and organ level phenotypes by Mavacamten in thin filament variants, we trialled additional pharmacological targets to establish the efficacy of directly targeting primary pathomechanisms in this subset of HCM carriers.

We tested on-market L-type calcium and late sodium current blockers^38^, as well as upregulation of sarcoplasmic endoplasmic reticular calcium ATPase (SERCA), as SERCA is reduced in human HCM samples^39–41^. The effect of late sodium current block on relaxation in *TNNT2*^R92Q/+^ and *TNNI3*^R21C/+^ cardiomyocytes was minimal, with partial correction of hypercontractility (Supplementary Fig. 3a,b). A similar trend was observed for L-type calcium current block (Supplementary Fig. 3c,d), although severe negative inotropic effects were present. SERCA upregulation restored tension relaxation, despite an undesirable further positive inotropic effect (Supplementary Fig. 3e,f). Only dual application of SERCA upregulation and 0.5 $\upmu$M Mavacamten achieved complete phenotype resolution (Supplementary Fig. 3e,f, triangles).

**Figure 6:**
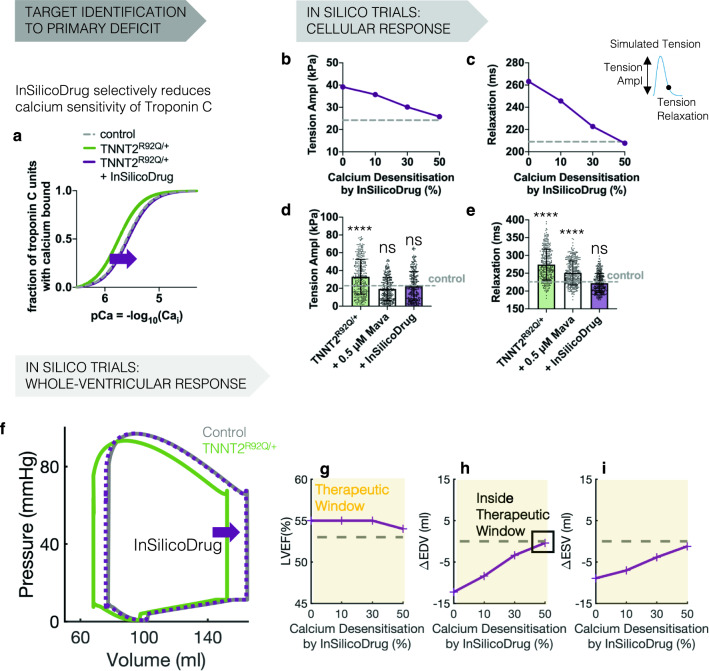
In-silico identification of sarcomere druggable targets for the resolution of the TNNT2^R92Q/+^ phenotype when Mavacamten’s action is suboptimal. (**a**) Based on identified pathophysiology, the in-silico designed drug should selectively reduce calcium sensitivity of troponin C and shift the steady state relationship between calcium bound to troponin and free calcium to the right. (**b**,**c**) Effect of the in-silico designed drug on tension amplitude (**b**) and relaxation (**c**) of a simulated TNNT2^R92Q/+^ cardiomyocyte. (**d**,**e**) Comparison of the effects of 0.5 $\upmu$M Mavacamten and the in-silico designed drug (50% calcium desensitisation of the thin filament) on the tension amplitude (**d**) and relaxation (**e**) of a population of simulated TNNT2^R92Q/+^ cardiomyocytes. Data presented as mean and SD. Significance with respect to untreated control (dotted grey lines) was tested with Kruskal–Wallis with post hoc Dunn correction (N = 348 for each in-silico population). (**f**) TNNT2^R92Q/+^ pressure–volume loop normalisation by the in-silico designed drug. (**g**–**i**) Dose-dependent effect of the in-silico designed drug on the LVEF (**g**), end-diastolic (**h**), and end-systolic (**i**) volume of the left ventricle of the virtual TNNT2^R92Q/+^ carrier. Yellow areas identify therapeutic windows. The in-silico identified drug restores all phenotype components within therapeutic window.

Clinical upregulation approaches are often complicated by delivery strategies, which would be further complicated by trialling a non-targeted polytherapeutic/polypharmacy approach by the addition of Mavacamten. We hypothesised that a single-target therapeutic could provide a more efficacious therapeutic strategy than a multi-target one. Therefore, we generated a framework to define a novel thin filament-specific drug. We took advantage of the finding that the diastolic dysfunction of *TNNT2*^R92Q/+^ and *TNNI3*^R21C/+^ variants are both due to increased myofilament calcium sensitivity. We tested an idealised pharmacological intervention specifically acting as a calcium desensitiser at the thin filament (Fig. 6a). This strategy dose-dependently normalised both contraction and relaxation in thin filament HCM (Fig. 6b,c), leading to phenotype resolution in simulated *TNNT2*^R92Q/+^ cardiomyocytes at a 50% calcium sensitivity reduction (Fig. 6d,e). A 50% decrease in calcium sensitivity at the organ level also restored both systolic and diastolic ventricular dysfunction without deleterious effects on LVEF (Fig. 6f,g). Both end-diastolic (Fig. 6h) and end-systolic (Fig. 6i) volumes were corrected within therapeutic window.

## Discussion

Within the field of genetic cardiomyopathy and more widely in cardiovascular research there have been many techniques developed to accelerate variant classification and therapeutic screening^22,42,43^, spanning biochemical investigations, rapid variant classification platforms, and hiPSC-CM-based systems. But with ever-growing genomic datasets, biological/biochemical phenomics is still a bottleneck in translation from bench to bedside. Herein we describe a synergistic hybrid biological and in-silico human system that accelerates hypothesis generation and testing in variant classification, pathomechanism discovery, and therapeutic targeting. Our validated human modelling and simulation approach bridges from disease-causing mutations to clinical disease manifestation. Our approach is advantageous as it does not need information on the biological/clinical phenotype. The only input needed is the mutation-specific biophysical defect/s which then allow the investigation of variants that do not display a clear phenotype. We address hiPSC-CMs immaturity through the modelling of human adult virtual cardiomyocytes, and physiological variability through populations of models. We scale this analysis to genotype-specific clinical phenotypes which are obtained through whole-ventricular simulations. We used human-based digital twins and mechanistic simulations to understand pathomechanisms in HCM and identify druggable targets. Altogether, this generates digital evidence of incomplete rescue of thin filament HCM phenotypes by Mavacamten but shows the promise of combination therapies or novel targeted therapeutics.

hiPSC-CMs provide a human cellular system to validate these findings^44,45^, as they capture patient-specific genotype–phenotype relationships and pharmacological responses^46^. hiPSC-CM experiments show that both thick (*MYH7*^R403Q/+^) and thin (*TNNT2*^R92Q/+^, *TNNI3*^R21C/+^) filament HCM variants cause cellular hypercontractility and diastolic dysfunction^22,26,27^, consistent with clinical findings in HCM^25,35,47^. Our simulations show that SRX destabilisation is central to hypercontractility in thick filament HCM variants in *MYH7*^22,42,48^, but cannot explain impaired relaxation. We found that, secondarily to myosin activation, knock-on thin filament activation prolongs calcium binding to troponin^33^, slowing cellular relaxation in the *MYH7*^R403Q/+^ phenotype. When applying these mechanisms to whole-ventricular simulations, the *MYH7*^R403Q/+^ ventricles presented lower end-diastolic and end-systolic volumes and increased LVEF, consistent with clinical findings^35,47^.

We found that the thin filament HCM variants studied here have changes in thin filament activation driven by altered calcium sensitivity^30^. *TNNT2*^R92Q/+^ sensitises myofilament calcium binding by alterations to cooperative activation^30^, where its initial biophysical insult could reside in tropomyosin positioning^29^. Our in-silico models confirm this and show indirect calcium sensitisation of the myofilament as a key determinant of diastolic dysfunction and calcium transient abnormalities in *TNNT2*^R92Q/+^^30,31,49^. This is confirmed by whole-ventricular simulations, where the increased myofilament calcium sensitivity drives a greater diastolic insufficiency in *TNNT2*^R92Q/+^ than in *MYH7*^R403Q/+^ ventricles.

*TNNI3*^R21C/+^ hiPSC-CMs exhibit faster calcium transient rise and prolonged transient decay, alongside hypercontractility and impaired relaxation^26^. We show that the abnormal calcium transient phenotype is caused by increased calcium binding and slowed dissociation from troponin, leading to greater crossbridge recruitment, hypercontraction, and prolonged relaxation^32^.

Mavacamten, an allosteric myosin ATPase inhibitor, was recently approved by the FDA for the treatment of symptomatic obstructive HCM. Developed to target the core molecular mechanisms that cause HCM^24^, Mavacamten reduces ATPase activity and increases myosin SRX formation across a variety of experimental models^22–24,48,50–54^. Building on positive preclinical results, Mavacamten was advanced to clinical testing^25,37,55^, but the exact mechanisms through which it restores contractile function, especially across HCM genotypes, are not fully understood. To investigate this, we extended our cellular human model to integrate modulation of myosin availability.

Application of Mavacamten to our *MYH7*^R403Q/+^ thick filament HCM models predicted a clear resolution of cellular HCM phenotypes, in agreement with our *MYH7*^R403Q/+^ hiPSC-CM data. Thin filament HCM was not fully rescued by Mavacamten, confirmed using *TNNT2*^R92Q/+^ and *TNNI3*^R21C/+^ hiPSC-CMs^22,26,27^. Mavacamten was able to dose-dependently suppress cellular contractility in-silico irrespective of genotype, but its efficacy in restoring diastolic function was diminished in thin filament variants. We provide digital evidence that this is because Mavacamten does not directly target the HCM causative mechanisms in these variants.

Mavacamten’s administration in ventricular simulations of thick filament genotype-positive phenotype-negative HCM reverses early phenotypic abnormalities of hyperdynamic ventricular contraction and impaired ventricular filling, with modest LVEF decrease. This suggests that Mavacamten can potentially be used as a treatment to prevent disease progression, in agreement with preclinical testing^24^. Our simulations predicted a tolerable decrease in LVEF at 350–700 ng/ml Mavacamten with detrimental reductions in LVEF at 1000 ng/ml, as previously reported^25,37^. Simulations show that hyperdynamic ventricular contraction is corrected by Mavacamten in thin filament variants. However, Mavacamten only showed correction of impaired ventricular filling in *MYH7*^R403Q/+^ and not *TNNT*^R92Q/+^. In agreement with our cellular results, whole-organ diastolic insufficiency caused by thin filament HCM cannot be corrected by Mavacamten without detrimental suppression of LVEF. Our study highlights the importance of future investigations into Mavacamten’s utility in specific HCM genotype populations.

We define the importance of a pharmacogenetic approach that defines and targets the incident mechanism of pathophysiology in thin filament HCM, which is an increased myofilament calcium sensitivity. We show this is feasible by selective calcium desensitisation, which restores cellular function and normalises whole-organ contractility without detrimental suppression of LVEF in *TNNT2*^R92Q/+^. This highlights the potential of hybrid human systems as powerful tools to accelerate the development and administration of rational therapies in a pathomechanism-specific manner.

### Study limitations

The integration of biophysical evidence, human cardiac pathophysiology, and in-silico modelling and simulation can accelerate discovery research, help to define HCM pathomechanisms, and aid in drug discovery. However, in-silico methodologies have intrinsic limitations. By design in-silico models are simplified representations of biological systems that cannot, at this point, account for their full complexity. In-silico predictions rely heavily on data input quality and model assumptions. It is only through the coupling with experimental models and biological data that we can ensure accurate model calibration and robust in-silico predictions, as conducted here. Nevertheless, in-silico methodologies provide a means to conduct mechanistic and systematic analyses that would not otherwise be possible. Coupling biological data with in-silico models additionally helps to overcome limitations of experimental systems. In this regard hiPSC-CMs are a useful tool for interrogating disease mechanisms in a human and patient-specific cell^46^. However, even in their most mature state they present highly variable structural and functional features falling short of the maturity of adult cardiomyocytes^56^. This is why we synergistically combined the strengths of the two systems to extend their mechanistic insight. It is through this iterative use of experimental data and model predictions that we can provide novel insights and generate novel hypotheses to better understand HCM.

Here we developed a phenomenological representation of myosin states, to model the effect of their adaptation in HCM and therapeutically by Mavacamten. This is only a simplified description of the biological system and does not account for the full complexity of the processes involved and their dynamics. Despite these simplifications, this model has successfully simulated the effects of HCM causing mutations and of a drug therapy that directly alter myosin SRX formation. This enabled to our knowledge the first simulations of Mavacamten’s pharmacological mechanism. This was carried out according to the most established hypothesis of Mavacamten’s function as a stabiliser of myosin SRX, reducing the number of myosin heads that can interact with actin. Other proposed mechanisms of action^57,58^ were not considered in this work but can be incorporated and tested in simulations should relevant data become available.

This study was designed to define primary pathomechanisms in HCM preceding compensatory responses that emerge during the course of the disease. For this reason, we did not incorporate long term effects of HCM mutations on cardiac electromechanical function, and we cannot draw conclusions about long-term effects of HCM variants on cellular, or organ level function. Understanding the connection between early and late disease stages is key to fully understanding the pathophysiology of HCM and define the therapeutic options that would be most effective for different disease stages. The hybrid biological in-silico framework presented here can be evolved to investigate disease progression as whole organ data becomes available.

## Methods

### Human in-vitro single cell model

Heterozygous pathogenic missense variants *TNNI3*^R21C/+^, *TNNT2*^R92Q/+^ and *MYH7*^R403Q/+^ that cause HCM were introduced using CRISPR/Cas9 technology as previously described in an hiPSC cell line harbouring GFP-labelled titin^59,60^. Monolayer differentiation of control and WT cell lines was performed via Wnt pathway modulation with small molecule inhibitors. Cells were induced to the mesodermal layer with 12 µM CHIR99021 in RPMI1640/B27 minus insulin for 24 h. This was considered to be day 0 of differentiation. At the end of 24 h, CHIR99021 was diluted to 6 µM with fresh RPMI1640/B27 minus insulin for a further 24 h. On day 2, media were replaced with RPMI1640/B27 minus insulin. On day 3, cells were induced to cardiac lineage specification with 5 µM IWP2 in RPMI1640/B27 minus insulin for 48 h. Cells were then cultured in the absence of insulin to day 10. Spontaneous contraction was observed on day 9–11 of differentiation. Cells were subjected to two 48-h rounds of metabolic selection starting on day 10, with glucose-free RPMI/B27 plus insulin. Cells were then cultured to day 24 in RPMI1640/B27 plus insulin. On day 24, cells were passaged onto glass-bottom dishes and cultured to day 28 in RPMI1640/B27 plus insulin. On day 28, cells designated for calcium phenotyping were induced to express the red genetically encoded calcium indicator for optogenetics, RGECO1, via adenoviral transduction at MOI 40 for 24 h. The next day, media were changed back to RPMI1640/B27 plus insulin. Plates designated for contractility measurement were cultured to day 30 in to RPMI1640/B27 plus insulin.

All measurements were prepared in triplicate technical replicates and triplicate biological replicates. Differentiations of separate cell passages were considered distinct biological replicates. Cells designated for contractility measurement were incubated for ten minutes at 37 °C/5% CO_2_ with the myosin inhibitor Mavacamten at 0.3, 1 and 3 µM prior to imaging in Tyrodes-HEPES buffer at 37 °C/5% CO_2_ and imaged directly. For calcium measurement, mutant and WT cells were equilibrated with Tyrode’s-HEPES buffer for ten minutes at 37 °C/5% CO_2_ and then imaged. Controls were provided by triplicate plates of equivalent concentrations of dimethylsulfoxide (DMSO) and triplicate plates incubated in the absence of any treatment to capture the mutant phenotype.

hiPSC-CMs from each group were imaged on an Olympus IX81 inverted microscope (Olympus, Japan) with a C-9100-13 EMCCD camera (Hamamatsu, Japan), under electrical stimulation of 20 V/1 Hz and at 37 °C. Videos were acquired at 50 fps, 560/25 nm excitation, 620/60 nm emission with a 565 nm dichroic mirror for calcium imaging and 50 fps, 485/20–25 nm excitation, 525/50 nm emission with a 495 nm dichroic mirror for contractility imaging. Contractility measurement was performed at 30 fps. A minimum of 30 cells were recorded per plate. Data was extracted with CalTrack^26^ and SarcTrack^61^ software.

### Human in-silico single cell model

We extended our coupled human electromechanical adult cardiomyocyte model^20,28,62^ to allow interrogation of how disease- and drug-induced changes in the physiological regulation of myosin function alter ventricular contractility. We hypothesised that changes in myosin SRX^63^ could be phenomenologically simulated with the coupled model through a modulation of actin-myosin crossbridge availability. This was computationally implemented as an explicit dependency of crossbridge formation on the proportion of myosin in the SRX state, which are inhibited and unable to bind actin, with respect to the rest of myosin heads that are available to drive contraction, which broadly exist in the myosin disordered relaxed state DRX. We introduced a new parameter $R = \frac{(DRX:SRX)}{{(DRX:SRX)}_{control}}$ into the cellular model to account for deviations from the control DRX:SRX ratio. We calibrated the modified cellular model to achieve changes in contractile function by SRX:DRX modulation that are consistent with experimental data. Details of the cellular model construction and calibration are reported in the supplement.

### Populations of in-silico human adult cardiomyocytes across different genetic backgrounds

From the extended control single cell model, a control population of healthy cardiomyocytes was built to consider physiological electromechanical variability^64^. An initial population of 2000 electromechanical models was created by sampling the fast and late sodium, transient outward potassium, rapid and slow delayed rectifier potassium, inward rectifier potassium, sodium-calcium exchanger, sodium–potassium pump, and the L-type calcium conductances, the sarcoplasmic reticulum calcium release flux, the calcium uptake via SERCA, calcium sensitivity of SERCA, intracellular sodium affinity of the sodium–potassium pump, calcium sensitivity of myofilament, and crossbridge cycling rates. Parameters were varied in the range [50–200]% of their baseline value with the Latin Hypercube Sampling technique. The population was calibrated based on action potential and calcium transient data as in Ref.^65^ and on active tension data (time to 50 and 95% transient decay). We considered experimental action potential, calcium transient, and active tension recordings data as in Ref.^20^.

The control population was then used to construct three populations with different genetic background, representative of the three HCM causing variants considered in this study.

### MYH7^R403Q/+^

To build the *MYH7*^R403Q/+^ population, we modelled the myosin ratio $R= \frac{(DRX:SRX)}{{(DRX:SRX)}_{control}}$ of 1.3, as measured experimentally in hiPSC-CMs^22^. To investigate the possible determinants of impaired cellular relaxation in MYH7^R403Q/+^, we compared simulation results obtained with this population, which only considered a change in R with results from a population which considered a positive feedback of myosin-based activation on thin filament activation (as described in the supplement).

### TNNT2^R92Q/+^ and TNNI3^R21C/+^

Different molecular and biophysical defects have been reported as primary drivers of cellular dysfunction in HCM caused by thin filament mutations. The *TNNT2*^R92Q/+^ mutation has been consistently reported to cause indirect calcium sensitization of the myofilaments^30,31^. Recently it was also shown that a primary molecular insult for the observed hypercontractility and consequent disease pathogenesis in *TNNT2*^R92Q/+^ is a mutation-induced alteration in tropomyosin positioning^29^. We conducted a sensitivity analysis to determine the independent contributions of calcium sensitisation or abnormal tropomyosin positioning to altered cellular contractility. The reverse rate constant that defines K_B_ (for tropomyosin positioning) in our baseline mechanical model and the parameter Ca50 that regulates calcium sensitivity of myofilaments were varied one at a time. K_B_ represents the equilibrium constant between the blocked and closed tropomyosin states. Specifically, parameters were varied in the range $\pm$ 30% of their baseline value. We computed the absolute sensitivities of active tension and calcium transient biomarkers to changes in the model parameters as in Ref.^34^. From the sensitivity results on the relative contribution of tropomyosin positioning and calcium buffering in determining abnormal calcium and contractility with the *TNNT2*^R92Q/+^ variant, an average mutant remodelling was selected (Ca50 × 0.7 and K_B_ × 0.8, consistent with the reported increase in contractility under this mutation) and applied to the control population to generate a *TNNT2*^R92Q/+^ population.

The *TNNI3*^R21C/+^ mutation increases calcium binding of cardiac troponin and the affinity of cardiac troponin C for cardiac troponin I in solution, showing increases in the calcium sensitivity of myofibril tension development, and a prolonged early slow phase of relaxation^32^. Building upon these results we considered abnormal calcium-troponin binding as the primary mutation-induced defect. We tested the contribution of changes in calcium sensitivity and dissociation rate from troponin to cellular contractility and calcium transient morphology by means of a sensitivity analysis. Specifically, the Ca50 parameter that describes the calcium sensitivity and the unbinding rate of calcium from troponin k_off_ were considered. Ca50 was varied in the range $\pm$ 30% of its baseline value, and k_off_ was varied in the range $\pm$ 50% of its baseline value. We computed the absolute sensitivities of active tension and calcium transient biomarkers to changes in the model parameters as in Ref.^34^. Similar to the troponin T variant, an average remodelling of calcium binding (Ca50 × 0.7) and dissociation (k_off_ × 0.5) from troponin under *TNNI3*^R21C/+^ was chosen so that it replicates the increase in contractility observed experimentally and was then applied to the control population to construct the *TNNI3*^R21C/+^ population.

### Human-based in-silico drug trials

We used our generated cellular populations to run in-silico trials of pharmacological therapies and identify effective targets based on identified pathomechanisms for each genotype. We simulated the novel myosin modulator Mavacamten by using the relationship we established between Mavacamten concentrations and myosin availability when we calibrated our cellular model (as explained in the supplement). This provided a dose–response curve through which values for the model parameter $R= \frac{(DRX:SRX)}{{(DRX:SRX)}_{control}}$ can be estimated from Mavacamten concentrations, enabling its testing in virtual trials. We simulated Mavacamten concentrations in the experimentally relevant range of 0.3–1.0 $\upmu$M^22,24,58^. We computed biomarkers of active contraction to determine the effect of the drug on the contractility and diastolic function of simulated *MYH7*^R403Q/+^, *TNNT2*^R92Q/+^, and *TNNI3*^R21C/+^ cardiomyocytes, and compared simulated results with experimental evidence^22,26,27^. Specifically, we considered the amplitude of the tension transient, and the relaxation time quantified as time to 90% transient decay of the simulated twitch tension.

In order to identify additional therapeutic targets that could aid in phenotype resolution for the troponin variants, we also tested the effect of additional pharmacological strategies. Specifically, we tested different levels of L-type calcium and late sodium currents block (20, 40, 60% block of current conductances) and different levels of SERCA upregulation (20, 50, 80% increase) in the presence and absence of 0.5 $\upmu$M Mavacamten. We computed biomarkers of active contraction to determine the effect of these simulated therapeutic strategies on the contractility and diastolic function of simulated *TNNT2*^R92Q/+^ and *TNNI3*^R21C/+^ cardiomyocytes.

Finally, we designed in-silico a pharmacological intervention that, through a direct calcium desensitisation of the myofilaments, could rescue the identified mechanisms of disease caused by the *TNNT2*^R92Q/+^ and *TNNI3*^R21C/+^ genetic variants, thereby restoring the associated disease phenotype. We tested different percentages (10, 30, and 50%) of calcium desensitisation by the in-silico identified drug on the cellular and organ function under *TNNT2*^R92Q/+^. We computed cellular and organ level mechanical biomarkers and compared therapeutic efficacy against Mavacamten.

### Human whole-ventricular electromechanical simulations

We conducted in-silico clinical trials of Mavacamten through human biventricular electromechanical simulations to investigate implications on clinical phenotypes.

We used a recently published biventricular model that features a torso-biventricular anatomy from clinical magnetic resonance imaging^21^. The torso-biventricular mesh features an average element edge length of 220 $\upmu$m. This results in a total of more than 1 million elements which require 360 cores and approximately 6 h in a supercomputer to simulate three heart beats of the baseline model. Computational cost thus prevents analysis of a population of models as presented for cellular data.

We computed pressure and volume transients and 12-lead electrocardiograms at clinically standardised lead locations as in Ref.^21^ and extracted clinical electrocardiograms biomarkers, end-diastolic and end-systolic volumes, and left and right ventricular ejection fractions.

Here we integrated our extended cellular electromechanical model, in control conditions and under the *MYH7*^R403Q/+^ and *TNNT2*^R92Q/+^ variants, to construct the digital twin of healthy and genotype-positive phenotype-negative HCM subjects. The scaling factor of cellular active tension T_scale_ in our biventricular model^21^ was calibrated to achieve 53% LVEF in control conditions, and set to 4. We conducted biventricular simulations as described in^21^ and studied how the changes in cellular contractile function driven by the *MYH7*^R403Q/+^- and *TNNT2*^R92Q/+^-specific remodelling affected whole-ventricular contractility.

To be able to conduct in-silico clinical trials of Mavacamten, we calibrated our cellular model of the drug with clinical data from healthy subjects as described in the supplement (Supplementary Fig. 4). Our calibrated model of Mavacamten allowed the estimation of the drug free plasma concentrations to be tested in-silico to simulate clinically administered drug doses. We used the calibrated model to test the dose-dependent effect of 500, 1000, and 1500 ng/ml Mavacamten (based on clinical trials^25,37,55^) on whole-ventricular electromechanical function. Simulated clinical biomarkers were compared with clinical evidence^25,37,55^.

### Software and stimulation protocols

Cellular simulations were conducted as in Ref.^20^ using MatLab (Mathworks Inc. Natick, MA, USA) and the ordinary differential equation solver ode15s. In each simulation, we delivered a stimulus current of − 53 $\upmu$A/$\upmu$F with 1 ms duration, and considered a fixed extension ratio ($\lambda$, sarcomere length over sarcomere length at rest) of 1. For each simulation, steady-state was reached at 1 Hz pacing before biomarkers were computed. Simulations of populations of models were conducted using the University of Oxford Advanced Research Computing (ARC) facility.

Whole ventricular simulations were conducted as in Ref.^21^, using the high-performance numerical software Alya for complex coupled multi-physics and multi-scale problems^66^ on the supercomputer *Piz Daint* of the Swiss National Supercomputing Centre. We simulated three beats of 800 ms cycle length with cellular models in steady-state conditions, and the pressure–volume loop and ECG convergence were assessed before computing clinically relevant endpoints.

### Statistical analyses

Statistical analyses were performed using GraphPad Prism version 8.4.3 for macOS (GraphPad Software, San Diego, CA, www.graphpad.com). Normality in all data sets, defined as alpha < 0.05, was assessed by the D’Agostino-Pearson normality test. Single comparisons of data modelled by a normal distribution was assessed by a double-tailed Student t test; multiple comparisons were conducted with a one-way ANOVA with a post hoc correction for the number of comparisons. Data that were not modelled by a normal distribution were assessed by a nonparametric Mann–Whitney test. When multiple comparisons were tested in unpaired data, Kruskal–Wallis was used with a post hoc Dunn correction. In all instances, a significance cut-off of P < 0.05 was used.

## Supplementary Information

Supplementary Information.

## Data Availability

Cellular model code and Alya executable and simulation input files required to replicate the simulated results of this study, and hiPSC-CM experimental datasets reported in this study will be made available upon request. Requests for materials should be addressed to christopher.toepfer@cardiov.ox.ac.uk or alfonso.bueno@cs.ox.ac.uk.
